# DNA Replication and Strand Asymmetry in Prokaryotic and Mitochondrial Genomes

**DOI:** 10.2174/138920212799034776

**Published:** 2012-03

**Authors:** Xuhua Xia

**Affiliations:** 1 Department of Biology and Center for Advanced Research in Environmental Genomics, University of Ottawa, 30 Marie Curie, P.O. Box 450, Station A, Ottawa, Ontario, Canada; 2 Ottawa Institute of Systems Biology, Ottawa, Canada

**Keywords:** Archaea, DNA replication, deamination, GC skew, mitochondria, mutation, origin of replication, selection.

## Abstract

Different patterns of strand asymmetry have been documented in a variety of prokaryotic genomes as well as mitochondrial genomes. Because different replication mechanisms often lead to different patterns of strand asymmetry, much can be learned of replication mechanisms by examining strand asymmetry. Here I summarize the diverse patterns of strand asymmetry among different taxonomic groups to suggest that (1) the single-origin replication may not be universal among bacterial species as the endosymbionts *Wigglesworthia glossinidia*, *Wolbachia* species, cyanobacterium *Synechocystis* 6803 and *Mycoplasma pulmonis* genomes all exhibit strand asymmetry patterns consistent with the multiple origins of replication, (2) different replication origins in some archaeal genomes leave quite different patterns of strand asymmetry, suggesting that different replication origins in the same genome may be differentially used, (3) mitochondrial genomes from representative vertebrate species share one strand asymmetry pattern consistent with the strand-displacement replication documented in mammalian mtDNA, suggesting that the mtDNA replication mechanism in mammals may be shared among all vertebrate species, and (4) mitochondrial genomes from primitive forms of metazoans such as the sponge and hydra (representing Porifera and Cnidaria, respectively), as well as those from plants, have strand asymmetry patterns similar to single-origin or multi-origin replications observed in prokaryotes and are drastically different from mitochondrial genomes from other metazoans. This may explain why sponge and hydra mitochondrial genomes, as well as plant mitochondrial genomes, evolves much slower than those from other metazoans.

## INTRODUCTION

DNA strand asymmetry refers to the differential distribution of nucleotides between the two DNA strands, e.g., one has more A or C than the other. This implies a violation of Chargaff’s parity rule 2 [1], i.e., A = T and C = G within each strand. Consequently, strand asymmetry is typically measured by nucleotide skews such as GC skew and AT skew [2-9], referred hereafter as S_G_ and S_A_:

(1) SG = GC Skew = G−CG+CSA = AT Skew=A−TA+T

**Fig. (1):**
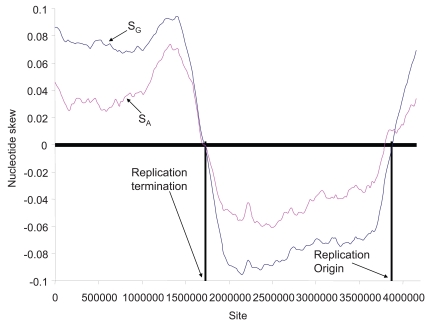
Nucleotide skew plot for the *Bacillus subtilis* genome (NC_000964), with window size = 537179 and step size = 21078. Each data point is at the beginning of its sliding window. The replication origin is identified as the genomic site where the GC skew (S_G_) changes from negative to positive and the replication termination is the site where S_G_ changes from positive to negative.

Chargaff’s parity rule 2 may be satisfied at the genomic level in spite of strong local strand asymmetry. For example, *Bacillus subtilis* studied by Chargaff and his colleagues [1] has its genomic nucleotide frequencies being 28.18%, 21.81%, 21.71%, and 28.30% for A, C, G and T, according to the genomic sequence deposited in GenBank (NC_000964). Thus, both S_G _and S_A_ are close to 0 (S_G_ = - 0.0021, S_A_ = 0.0023). However, *B. subtilis *genomic DNA exhibits strong local asymmetry (Fig. **1**). The asymmetry differs between the leading and the lagging strands, with the leading strand having more G than C and the lagging strand more C than G [2]. The strand compositional asymmetry is strong enough to allow the identification of the bacterial origin of replication (Fig. **1**) whose flanking sequences change direction in GC skew [2, 10-14] or in the components of the Z-curve [11, 15-17]. For this reason, strand asymmetry is often computed locally instead of globally, with the nucleotide skews computed with a sliding window. The validity and effectiveness of the *in silico *methods using strand asymmetry to identify the origin of replication in prokaryotic species are well established by many experimental verifications of the predicted replication origins [18] and the utility of these methods in practice has been demonstrated by many recent studies on prokaryotic genomes [15, 17, 19-24], mitochondrial genomes [25-28] and plasmid genomes [16].

**Fig. (2):**
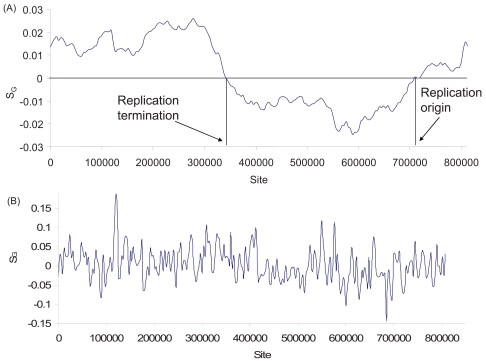
S_G_ plot for the *Mycoplasma pneumoniae* genome (NC_000912), with window size = 136694 and step size = 4081 (**A**), and with window size = 4000 and step size = 3000 (**B**).

The nucleotide skews in Eq. (1) were extended in two ways. The first leads to the cumulative skew [8] which is based on summation of adjacent skew values and is equivalent to nucleotide skews with a wider sliding window than what is used to compute individual skew values. For example, the *Mycoplasma pneumoniae *genome has been used to illustrate the advantage of the cumulative skew method which detects the replication origin while the original GC skew method does not [8]. The real difference is not really between the two methods, but between two different widths of the sliding window (Fig. **2**). A wide sliding window detects a clear change in polarity of strand asymmetry (Fig. **2A**), but a narrow window fails to (Fig. **2B**). In this review, the window size for skew plots is optimized with the criterion that the autocorrelation between the GC skew values of neighboring sliding windows is maximized. This method is implemented in DAMBE [29, 30].

The second extension of the nucleotide skews is to the word or motif skew [31] which is defined as

(2) $$S_{m}=\frac{N_{m}-N_{m_{\mathrm{rc}}}}{N_{m}+N_{m_{\mathrm{rc}}}}$$

where m is either a nucleotide (e.g., G or A) or a motif (e.g., ACG), m_rc_ is the reverse complement of m (m_rc_ = C if m = G, or m_rc_ = CGT if m = ACG), and N_x_ is the number of x in the sliding window (where x is either m or m_rc_). GC skew and AT skew are special cases of S_m_ when m is equal to either G or A, respectively, i.e., GC Skew is S_G_ and AT skew is S_A_. It is for this reason that I have denoted GC Skew and AT Skew by S_G_ and S_A_, respectively, in Eq. (1). 

While transcription is known to contribute to strand asymmetry [32, 33], the most important contributor to strand asymmetry is DNA replication associated with differential strand-specific mutation bias [21, 22, 34], which is confirmed by a study that assesses the contribution of both transcription and replication to strand asymmetry [23]. Because different replication mechanisms often lead to different patterns of strand asymmetry, much can be learned of replication mechanisms by examining strand asymmetry. In this review I will summarize the different patterns of strand asymmetry in different prokaryotic and mitochondrial genomes as a basis to infer the mechanism of DNA replication that gives rise to the diversity of strand asymmetry patterns. Based on the empirical evidence, I argue that (1) the common assumption of the single-origin DNA replication in bacterial species may not be valid because bacterial genomes from the endosymbionts *Wigglesworthia glossinidia* and *Wolbachia* (from *Drosophila melanogaster*) exhibit patterns of strand asymmetry strongly indicative of multiple origins of replication, (2) different replication origins in some archaeal genomes leave quite different patterns of strand asymmetry, suggesting that different replication origins in the same genome may be differentially used, (3) the pattern of strand asymmetry from mammalian mitochondrial genomes is consistent with the strand-displacement model of replication well documented in mammalian mitochondria [35-40], and this pattern is shared among mitochondrial genomes from representative vertebrate species, suggesting a similar DNA replication mechanism among vertebrate mitochondrial genomes, and (4) primitive forms of metazoans such as sponge and hydra, as well as plants, have mitochondrial strand asymmetry patterns similar to prokaryotes and drastically different from higher metazoans, suggesting that mitochondrial genomes in plants and in primitive invertebrate such as sponge and hydra share the a similar replication mechanism as their bacterial ancestor with a much lower replication error rate than that in mammalian mitochondrial genomes whose strand-displacement replication is highly error-prone. This sheds light on why mitochondrial genomes from mammals evolve much faster than those from sponge, hydra and plants.

## DNA REPLICATION AND STRAND ASYMMETRY IN PROKARYOTIC GENOMES

It is generally assumed that bacterial genomes have a single origin of replication [41, 42] whereas archaeal genomes tend to have multiple origins of replication [43, 44]. However, experimental verification of the exact number of replication origins is difficult and only a handful of prokaryotic species have their replication origins experimentally verified. Comparison of strand asymmetry patterns can shed lights on different replication mechanisms because different types of DNA replication typically lead to different patterns of strand asymmetry. 

## BACTERIAL GENOMES

**Fig. (3):**
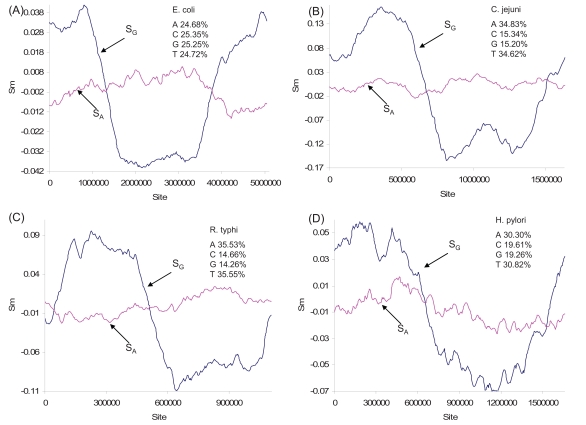
Nucleotide skew plots for the genomes of (**A**) *Escherichia coli* UTI89 (NC_007946, window size = 773338 and step size = 25328), (**B**) *Campylobacter jejuni* (NC_002163, window size = 251018 and step size = 8207), (**C**) *Rickettsia typhi wilmington* (NC_006142, window size = 191456 and step size = 5557) and (**D**) *Helicobacter pylori* (NC_000915, window size = 296433 and step size = 8339). Genomic nucleotide frequencies are shown for each species.

Many studies have documented strand asymmetry in eubacterial genomes associated with their single-origin mode of genome replication [2, 9, 45-47]. In general, there is an excess of G in the leading strand in many prokaryotic genomes examined [8, 17, 48-51], with the bias generally attributed to strand-biased deamination of C to U or m^5^C to T [9, 45, 52-54]. However, the distributions of nucleotides A and T along the leading and the lagging strands are much less consistent (Fig. **3**) as has been documented before [17]. For this reason, S_G_ has been used much more frequently in *in silico *identification of the replication origin and termination than S_A_.

In general, the pattern of S_G_ is highly consistent with the single-origin replication across a diverse array of bacterial species. This has led to the common assumption that all bacterial genomes replicate with a single origin. The assumption is reinforced by the strong conservation of the molecular machinery for bacterial DNA replication. For example, the DNA replication initiation factor DnaA protein from a marine cyanobacterium (*Prochlorococcus marinus* CCMP1375) can specifically recognize the chromosomal origin of replication (*oriC*) of both *E. coli* and *B. subtilis* [55]. Thus, given that many bacterial genomes are known to replicate with a single origin of replication, and that all bacterial genomes may be replicated the same way, it is natural for us to assume that all bacterial genomes replicate with a single origin of replication.

**Fig. (4):**
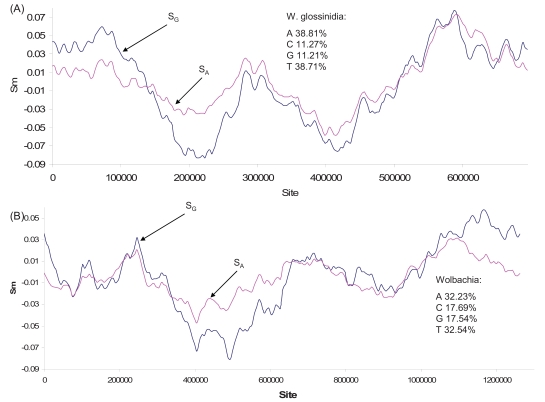
Nucleotide skew plots for the genome of (**A**) *Wigglesworthia glossinidia* (NC_004344), with window size = 89186 and step size = 3488, and (**B**) *Wolbachia* endosymbiont (NC_002978) of *Drosophila melanogaster*, with window size = 167632 and step size = 6338.

**Fig. (5):**
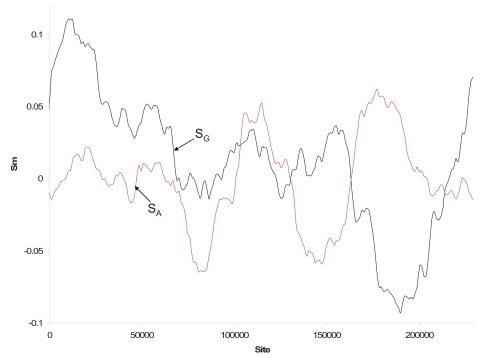
Nucleotide skew plot for the yeast (*Saccharomyces cerevisiae*) chromosome 1 (NC_001133), with window size = 29463 and step size = 1151.

The pattern of strand asymmetry in Fig. (**3**), however, is not universal among bacterial species (Fig. **4**). The possibility of multiple origins of replication is particularly strong in the AT-rich genome of two endosymbionts: *Wigglesworthia glossinidia* in tse-tse flies (*Glossina brevipalpis*) and *Wolbachia *in *Drosophila melanogaster* (Fig. **4**). The nucleotide skew plots with multiple changes of polarity are similar to that for the yeast (*Saccharomyces cerevisiae*) chromosome 1 replicated with multiple origins of replication (Fig. **5**). Thus, the assumption of single-origin replication in bacteria [41, 42] may be questionable.

There is no strong theoretical reason against some bacterial species having multiple origins of replication, other than the probably far-fetched possibility that daughter genomes arising from multiple origins of replication may fail to segregate properly into the two daughter cells. *Escherichia coli* genomes with an additional *oriC* inserted about 1 Mb apart from the regular *oriC* position seem to replicate normally, with both replication origins functioning identically and with no detectable difference in generation time or cell morphology from the wild-type cells [56]. This implies that, if mutation leads to the creation of an additional ectopic replication origin in an *E. coli *cell, there may be no strong selection against the mutant.

While multiple origins of replication typically would lead to multiple changes in polarity in the nucleotide skew plot, one should be careful in inferring multiple origins of replication based only on the observation of multiple changes in polarity in the nucleotide skew plots, because multiple changes in polarity can result from a variety of factors. For example, horizontal gene transfer is frequent in bacterial species, and a horizontally transferred sequence segment is likely to have quite different strand asymmetry patterns from the host genome, leading to additional changes in polarity in the skew plots. In other words, multiple changes in polarity in the skew plots may not result from multiple origins, but may instead result in the recent incorporation of multiple horizontally transferred genes. Similarly, there might be heterogeneity in strand asymmetry among different genes. For example, RNA genes typically form extensive secondary structure in which stems are double stranded and requires A=T and C=G (except for cases of U/G pairs in RNA). This implies that RNA genes should have different strand asymmetry patterns than the rest of the genomes, leading to additional changes in polarity in the skew plot. Also, if an rRNA gene cluster is duplicated in the opposite strand (which is the case for *Wigglesworthia glossinidia*), and if the rRNA is highly conserved (which is also true in *W. glossinidia*), then the recipient strand will have an irregular skew value at the position of the new rRNA genes. 

To alleviate these potential problems, I have generated the skew plots that included or excluded the protein-coding and rRNA genes. Such treatments do not alter the pattern of nucleotide skews in Fig. (**4**). While the pattern in S_G_ is indicative of multiple origins of replication (Fig. **4**), it is difficult to exclude alternative explanations. If genes switch strands frequently, then the strand asymmetry will be weak with multiple shallow peaks/valleys. This problem is particularly relevant to Wolbachia because of its mosaic genomic structure resulting from extensive recombination. My point is to highlight what is unresolved for future studies.

**Fig. (6):**
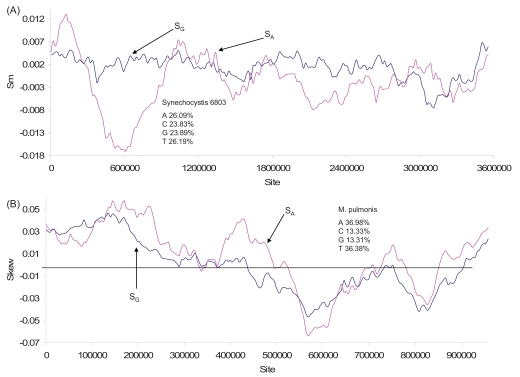
Nucleotide skew plots for the genome of (**A**) the cyanobacterium *Synechocystis* 6803 (NC_000908), with window size = 436362 and step size = 17867 and (**B**) *Mycoplasma pulmonis* (NC_002771), with window size = 142301 and step size = 4819.

In the cyanobacterium *Synechocystis sp.* 6803, S_G_ exhibits no recognizable change of polarity for any width of the sliding window. Its *dnaA *gene is located at sites 1350236..1351579 where no change in polarity of the strand asymmetry was observed in nearby sequence regions (Fig. **6A**). While S_A_ decreases and increases dramatically (Fig. **6A**), its change is typically not indicative of the origin of replication. The nucleotide skew plot in Fig. (**6A**) does not favor the hypothesis that the *Synechcystis sp.* 6803 genome has a single origin of replication that is fired consistently in all genome replications. 

The nucleotide skew plots for the AT-rich *Mycoplasma pulmonis *genome (Fig. **6B**) also do not suggest a single origin of replication because of multiple S_G_ changes in polarity. Instead of a sharp change in polarity, there is a long stretch of the genome with S_G_ values hovering above and below the zero line (Fig. **6B**). The genome contains many putative DnaA boxes [57], which is expected given the AT-richness of the genome. The genome is also peculiar in that a plasmid carrying an *oriC* would, after only a few passages, integrate into the predicted genomic *oriC* region [57]. This could lead to multiple origins of replication clustered together, with each having the potential to fire during genome replication. Such a hypothesis would potentially explain why there is a long stretch of genomic DNA with S_G_ values close to zero (Fig. **6B**), i.e., no strand asymmetry can be established within genomic regions with closely spaced multiple replication origins. 

The bacterial *oriC* is AT-rich and is expected to occur more frequently in AT-rich genomes. This suggests that AT-rich genomes have a greater tendency to harbor multiple origins of replication than GC-rich genomes. In this context, it is interesting to note that the bacterial species with a strong multi-origin replication signature in their strand asymmetry patterns, i.e., *Mycoplasma pulmonis, Wigglesworthia glossinidia* and *Wolbachia* are highly AT-rich genomes.

What bacterial genome would benefit from having multiple origins? If the genome is extraordinarily long, if the replication process is slow, or if the replication machinery (DNA-replication initiation and elongation proteins and enzymes) can be produced cheaply in multiple copies, then multiple replication origins would seem beneficial. Genomic data are available to address such a question.

Another point worth making in bacterial nucleotide skew plots is the diversity in the relationship between S_G_ and S_A_ (Figs. **1-4**, **6**). This diversity is unexpected given the common proposal that the main contributor to strand asymmetry is the strand-biased deamination of C to U or m^5^C to T during DNA replication [9, 45, 52-54]. If the strand asymmetry is maintained mainly by the C→U/T mutations, then we expect a negative relationship between S_G_ and S_A_, because reductions in C and increases in T will cause both an increase in S_G_ and a decrease in S_A_. Such a negative correlation is indeed observed in *Buchnera aphidicola *genome (not shown), but a strong positive correlation between S_G_ and S_A_ is also observed (e.g., all genomes in the genus *Bacillus*). Such a positive correlation cannot be explained by the pure C→U/T mutation bias [24, 58].

## ARCHAEAL GENOMES

**Fig. (7):**
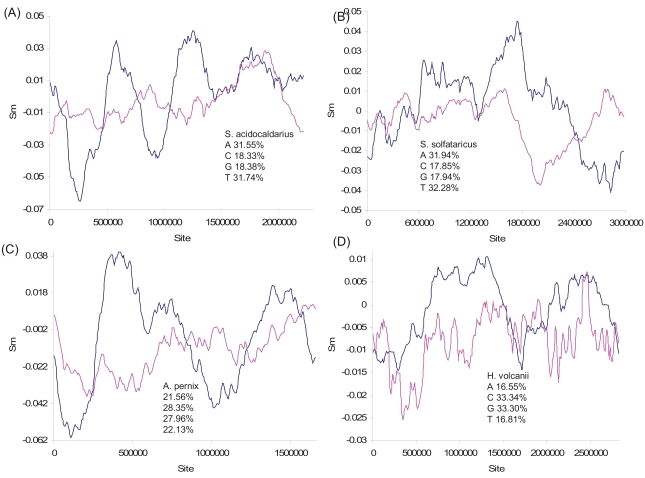
Nucleotide skew plots for genomes of (**A**) *Sulfolobus acidocaldarius* (NC_007181, window size = 317575, step size = 11129), (**B**) *Sulfolobus solfataricus* (NC_002754), window size = 413369, step size = 14961), (**C**) *Aeropyrum pernix* (NC_000854, window size = 238220, step size = 8348), and (**D**) *Haloferax volcanii* (NC_013967), window size = 405353 and step size = 14238. The species also contains three smaller replicons whose nucleotide skew plots are not shown).

Multiple replication origins are typically assumed for archaeal genome replication [43, 44, 59]. Multiple origins of replication implies multiple changes in polarity in nucleotide skew plots, which is well exemplified by several archaeal species with experimentally verified multiple origins of replication (Fig. **7**). *Sulfolocus salfataricus *and *S. acidocaldarius* both have three origins of replication [60, 61]. It is noteworthy that the S_G_ curve in *S. acidocaldarius *(Fig. **7A**) has valleys of different depths, similar to that for the yeast chromosome 1 (Fig. **5**). These valleys of different depths suggest that some replication origins are fired more frequently than others, leading to stronger strand asymmetry than other replication origins. In eukaryotes, different replication origins are not used synchronously or equally frequently [62]. This may also be true for archaeal replication origins. Differential usage of different replication origins has been documented in *Haloferax volcanii *[63]. In any case, the S_G_ pattern in Fig. (**7A**) casts doubt on the claim that the three replication origins in *Sulfolocus *species fire synchronously in each cell cycle [61]. 

The genome of *Aeropyrum pernix* contains two verified origins of replication, which is consistent with the S_G_ plot (Fig. **7C**). The different peaks and valleys again suggest different firing frequencies of different origins of replication. The two origins share some homology with two of the three replication origins in *Sulfolocus *species [42]*.* This raises the question of how *Sulfolocus *species acquired their third replication origin, i.e., whether it arose by accumulated mutations in the genome or whether it is acquired by capturing extrachromosomal element. The finding of a viral integrase element near the replication origins lends support for the latter [42].

**Fig. (8):**
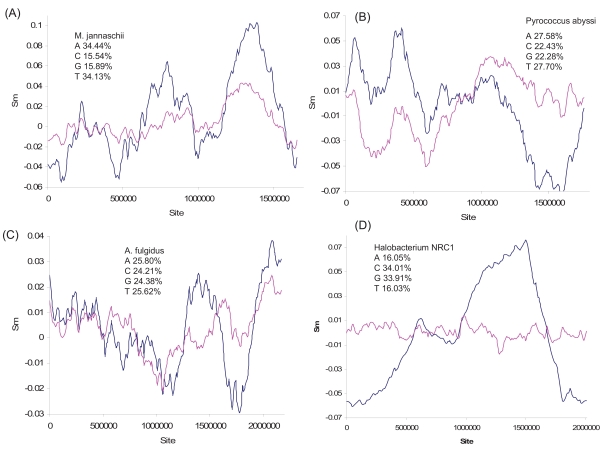
Nucleotide skew plot for genomes of (**A**) *Methanococcus jannaschii* (NC_000909, window size = 213047 and step size = 8324), (**B**) *Pyrococcus abyssi* (NC_000868, window size = 225116, step size = 8825), (**C**) *Archaeoglobus fulgidus* (NC_000917, window size = 299976, step size = 10892), and (**D**) *Halobacterium* NRC1 (NC_001133, window size = 307668 and step size = 10071).

The main chromosome of the halophilic archaeon *Haloferax volcanii* (which also has three smaller replicons) contains two origins of replication [63], which is also suggested by the two major changes in polarity in the S_G_ plot (Fig. **7D**). The origin of replication has not been identified in the *Methanococcus jannaschii* genome, but the multiple changes in polarity in the S_G_ plot (Fig. **8A**) from the genome strongly suggest multiple origins of replication. The genome also exhibits multiple peaks and valleys in marker frequency distributions [64], consistent with the interpretation of multiple origins of replication. The shared feature of multiple replication origins among these taxonomically diverse archaeal species suggests that multi-origin replication is the norm in Archaea.

Previous studies suggest only a single origin of replication in the genomes of three archaeal species: *Pyrococcus abyssi *[65, 66], *Archaeoglobus fulgidus* [64], and *Halobacterium* NRC1 [67]. While the S_G_ plot of *Halobacterium* NRC1 is consistent with a single-origin replication (Fig. **8D**), the S_G_ plot for *A. fulgidus *has two peaks, suggesting two putative replication origins. 

## DNA REPLICATION AND STRAND ASYMMETRY IN MITOCHONDRIAL GENOMES

Mitochondrial DNA (mtDNA) replication has been studied most thoroughly in mammals. Mammalian mtDNA has two strands of different buoyant densities and consequently named the H-strand and the L-strand. The two strands have different nucleotide frequencies, with the H-strand rich in G and T and the L-strand rich in A and C, which strongly affects the codon usage of genes on the two strands [28]. This strand asymmetry can be well explained by the strand-displacement model of mtDNA replication [35-40].

During mtDNA replication, the L-strand is first used as a template to replicate the daughter H-strand, starting at the origin of replication O_H_, while the parental H-strand was left single-stranded for an extended period because the complete replication of mtDNA takes nearly two hours [35-37]. After about 2/3 of the daughter H-strand has been synthesized and the second origin of replication (O_L_) is exposed, the parental H-strand is used as a template to synthesize the daughter L-strand. Thus, different parts of the H-strands are in single-stranded form for different periods of times.

Spontaneous deamination of both A and C [52, 53] occurs frequently in human mtDNA [68]. Deamination of A leads to hypoxanthine that pairs with C, generating an A/T→G/C mutation. Deamination of C leads to U, generating C/G→U/A mutations. Among these two types of spontaneous deamination, the C→U mutation occurs more frequently than the A→G mutation [53]. In particular, the C→U mutation mediated by the spontaneous deamination occurs in single-stranded DNA more than 100 times as frequent as double-stranded DNA [54]. Note that these C→U sites will immediately be used as template to replicate the daughter L-strand, leading to a G→A mutation in the L-strand after one round of DNA duplication. Such mutation patterns are expected to leave their footprints on different parts of the H-strands left single-stranded for different periods of time.

**Fig. (9):**
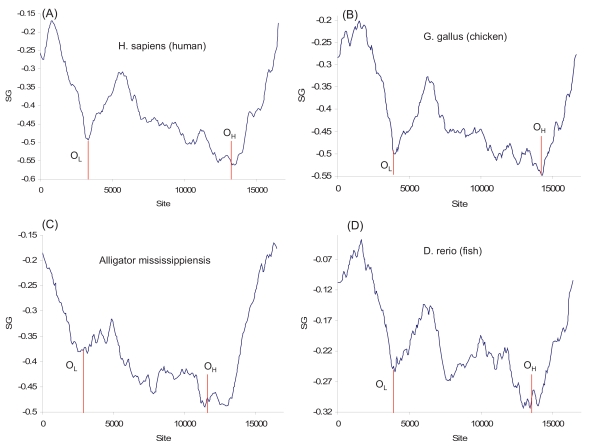
S_G_ plots for the L-strand of the mitochondrial genomes of (**A**) *Homo sapiens* (NC_012920), (**B**) *Gallus gallus* (NC_001323), (**C**) *Alligator mississippiensis* (NC_001922), and (**D**) *Danio rerio* (NC_002333). Inferred locations of the two replication origins (O_H_ and O_L_) are indicated.

While experimental evidence for the strand-displacement model is limited to mammalian species, the nearly identical pattern of strand asymmetry among representative vertebrate species (Fig. **9**) suggests that the replication mechanism is most likely shared. The reduction in S_G_ correspond to the reduction of C in the H strand (and the associated G in the L strand), allowing us to infer the location of replication origins O_H_ and O_L_ (Fig. **9**).

**Fig. (10):**
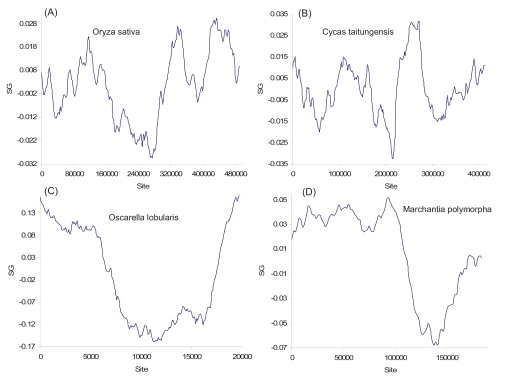
S_G_ plots for mitochondrial genomes of (**A**) *Oryza sativa* (NC_007886), (**B**) *Cycas taitungensis* (NC_010303), (**C**) the sponge *Oscarella lobularis* (NC_014863), and (**D**) the liverwort *Marchantia polymorpha* (NC_001660).

The pattern of strand asymmetry among mitochondrial genomes in vertebrate species is dramatically different from those of prokaryotic species or the yeast (Figs. **1-8**). In particular, the S_G_ values for the vertebrate species are all negative (and would be all positive for the complementary strand), in contrast to the S_G_ values of prokaryotic species which fluctuate above and below the zero line. This suggests not only local strand asymmetry, but also global strand asymmetry in vertebrate mitochondrial genomes. This is confirmed by the genomic S_G_, computed from genomic C and G frequencies from representative vertebrate mitochondrial genomes (Table **1**). Invertebrate mitochondrial genomes also exhibit consistent and strong global strand asymmetry (Table **1**), except for the most primitive ones such as the sponge (*Oscarella lobularis*) and the hydra (*Hydra oligactis*), representing Porifera and Cnidaria, respectively. The sponge and hydra mtDNAs have S_G_ values similar to those in plant mtDNA. The two animal groups they represent are also similar to plants in having slower evolutionary rates in their mtDNA than in their nuclear genomes [69], in contrast to other metazoans whose mtDNA evolves much faster than their nuclear genomes. As evolutionary rate is largely determined by mutations introduced during DNA replication, one would expect that mtDNA in plants and in primitive invertebrates such as Porifera and Cnidaria should have DNA replication different from the strand-displacement model established for mammalian mtDNA. The nucleotide skew plots (Figs. **9**, **10**) are consistent with this suggestion.

The pattern of mtDNA strand asymmetry in higher plants (e.g., *Oryza sativa* and *Cycas taitungensis*), as characterized by the S_G_ plots (Fig. **10A-B**), suggests multiple origins of replication with the S_G_ curve sharply crossing the zero line multiple times. This is similar to those observed in eukaryotic nuclear genomes or in archaeal genomes with multiple replication origins. Interestingly, for primitive forms of plants such as the liverwort *Marchantia polymorpha*, or primitive forms of metazoans such as the sponge *Oscarella lobularis,* the pattern of strand asymmetry (Fig. **10 C-D**) is indistinguishable from what is typically seen in bacterial genomes with a single origin of replication. The S_G_ plot of the *Hydra oligactis* mitochondrial genome is similar to that of *Oscarella lobularis* except for a slightly more pronounced secondary peak. All these patterns of strand asymmetry is dramatically different from those observed in vertebrate mtDNA (Fig. **9**) and may explain the extremely slow rate of evolution between plants/sponge and higher metazoans. In other words, mitochondrial genomes in plants and primitive invertebrates may maintain the high-fidelity replication in their bacterial ancestor, whereas the error-prone strand-displacement replication evolved, likely as a secondary consequence of some advantageous traits, in a lineage leading to vertebrate mitochondrial genomes. The diversification of mtDNA replication mechanisms has not been thoroughly explored in the context of evolution.

In summary, patterns of strand asymmetry are diverse among different taxonomic groups and can tell us much about the molecular mechanism of DNA replication. The single-origin replication may not be universal among bacterial species as the endosymbionts (*Wigglesworthia glossinidia*, and *Wolbachia* species), the cyanobacterium *Synechocystis* 6803, and *Mycoplasma pulmonis* all have their genomes exhibiting strand asymmetry patterns consistent with the multi-origin mode of replication. Different replication origins in some archaeal genomes leave quite different patterns of strand asymmetry, suggesting that different replication origins in the same genome may be differentially used. Vertebrate species share one strand asymmetry pattern consistent with the strand-displacement replication documented in mammalian mtDNA, suggesting that the mtDNA replication in mammals may be universal among vertebrates. Mitochondrial genomes from primitive forms of metazoans such as the sponge and hydra, as well as those from plants have strand asymmetry patterns similar to the single-origin or multi-origin types of DNA replication observed in prokaryotes. This may explain why sponge and hydra mtDNA, as well as plant mtDNA, evolves much slower than other metazoan mtDNA.

I should finally emphasize the importance of using statistical criteria when referring to peaks or changes in polarity in the skew plots. Take S_G_ for example, the standard deviation has been formulated as [2]:

(3) $$S_{S_{G}}=\frac{2}{C+G}\sqrt{\frac{\mathrm{CG}}{C+G}}$$

A peak in the S_G_ plot therefore refers specifically to a peak that protrude above the line of mean S_G_+1.96s, and a valley below the line of mean S_G_-1.96s, assuming the 0.05 significance level and that the window is sufficiently wide for the distribution of S_G_ approximating the normal distribution. I encourage all programmers to include the 95% confidence intervals for nucleotide or word skew plots. 

## Figures and Tables

**Table 1:** Nucleotide Frequencies (P_A_, P_C_, P_G_ and P_T_) and GC bias (S_G_) for Representative Metazoans and Plants. Note that S_G_ of the Complementary Strand has the Same Value but a Different Sign

Species	Accession	Length	P_A_	P_C_	P_G_	P_T_	S_G_
Oscarella lobularis	NC_014863	20260	0.333	0.176	0.173	0.318	-0.006
*Hydra oligactis*	NC_010214	16314	0.348	0.114	0.124	0.414	0.039
*Caenorhabditis elegans*	NC_001328	13794	0.314	0.089	0.149	0.448	0.253
*Schistosoma japonicum*	NC_002544	14085	0.249	0.084	0.206	0.462	0.422
*Drosophila melanogaster*	NC_001709	19517	0.418	0.103	0.076	0.404	-0.150
*Ciona intestinalis*	NC_004447	14790	0.342	0.095	0.119	0.444	0.116
*Branchiostoma lanceolatum*	NC_001912	15076	0.269	0.159	0.214	0.358	0.148
*Eptatretus burgeri*	NC_002807	17168	0.328	0.229	0.106	0.337	-0.366
*Mitsukurina owstoni*	NC_011825	17743	0.323	0.254	0.134	0.290	-0.309
*Danio rerio*	NC_002333	16596	0.319	0.239	0.160	0.281	-0.198
*Xenopus laevis*	NC_001573	17553	0.331	0.235	0.135	0.300	-0.270
*Alligator mississippiensis*	NC_001922	16646	0.312	0.295	0.135	0.257	-0.371
*Gallus gallus*	NC_001323	16775	0.303	0.325	0.135	0.238	-0.412
*Mus musculus*	NC_005089	16299	0.345	0.244	0.124	0.287	-0.328
*Marchantia polymorpha*	NC_001660	186609	0.285	0.210	0.214	0.291	0.009
*Cycas taitungensis*	NC_010303	414903	0.264	0.235	0.235	0.266	0.000
*Arabidopsis thaliana*	NC_001284	366924	0.279	0.225	0.222	0.273	-0.006
*Oryza sativa indica*	NC_007886	491515	0.279	0.219	0.220	0.283	0.002
*Sorghum bicolor*	NC_008360	468628	0.281	0.220	0.217	0.282	-0.008
*Triticum aestivum*	NC_007579	452528	0.279	0.221	0.222	0.278	0.002

## APPENDIX 1

How to generate nucleotide skew plots in DAMBE

1. Download and install DAMBE which is freely available at http://dambe.bio.uottawa.ca/dambe.asp
2. Download any genomic sequence that you wish to generate nucleotide skew plots from, e.g., *E. coli *K12 genome NC_010473 from GenBank and save to your computer, say C:\data\EcoliK12.gbk. Alternatively, you can use sequence files already on your computer.
3. Start DAMBE, click ‘File|Open standard sequence file’. Browse to C:\data and open the ‘EcoliK12.gbk’ file.
4. In the ensuing ‘Process GenBank File’ dialog, the default is ‘Whole sequence’. Keep the default and click the ‘OK’ button.
5. In the next dialog, the default is ‘Non-protein nuc. seq’. Keep the default and click the ‘Go’ button. The sequence will be displayed
6. Click ‘Seq.Analysis|Genome|GC Skew’. In the ensuing dialog, check the ‘Circular genome’ checkbox and click ‘Go’ button.
7. Two plots will be generated, one for S_G_ and one for S_A_. The window-specific data underlying the plots are also displayed.
